# Directed evolution driving the generation of an efficient keratinase variant to facilitate the feather degradation

**DOI:** 10.1186/s40643-022-00524-4

**Published:** 2022-04-04

**Authors:** Jing Zhang, Chang Su, Xiao-Li Kong, Jin-Song Gong, Yan-Lin Liu, Heng Li, Jiufu Qin, Zheng-Hong Xu, Jin-Song Shi

**Affiliations:** 1 Key Laboratory of Carbohydrate Chemistry and Biotechnology, Ministry of Education, School of Life Sciences and Health Engineering, Jiangnan University, Lihu Avenue No. 1800, Wuxi, 214122 People’s Republic of China; 2 National Engineering Research Center for Cereal Fermentation and Food Biomanufacturing, School of Biotechnology, Jiangnan University, Wuxi, 214122 People’s Republic of China; 3 Jiangsu Provincial Engineering Research Center for Bioactive Product Processing, Jiangnan University, Wuxi, 214122 People’s Republic of China; 4 Science Center for Future Foods, Jiangnan University, Wuxi, 214122 People’s Republic of China

**Keywords:** Keratinase, Error-prone PCR, Feather wastes, Biodegradation, Feed additive

## Abstract

Keratinases can specifically degrade keratins, which widely exist in hair, horns, claws and human skin. There is a great interest in developing keratinase to manage keratin waste generated by the poultry industry and reusing keratin products in agriculture, medical treatment and feed industries. Degradation of keratin waste by keratinase is more environmentally friendly and more sustainable compared with chemical and physical methods. However, the wild-type keratinase-producing strains usually cannot meet the requirements of industrial production, and some are pathogenic, limiting their development and utilization. The main purpose of this study is to improve the catalytic performance of keratinase via directed evolution technology for the degradation of feathers. We first constructed a mutant library through error-prone PCR and screened variants with enhanced enzyme activity. The keratinase activity was further improved through fermentation conditions optimization and fed-batch strategies in a 7-L bioreactor. As a result, nine mutants with enhanced activity were identified and the highest enzyme activity was improved from 1150 to 8448 U/mL finally. The mutant achieved efficient biodegradation of feathers, increasing the degradation rate from 49 to 88%. Moreover, a large number of amino acids and soluble peptides were obtained as degradation products, which were excellent protein resources to feed. Therefore, the study provided a keratinase mutant with application potential in the management of feather waste and preparation of protein feed additive.

**Graphical Abstract:**

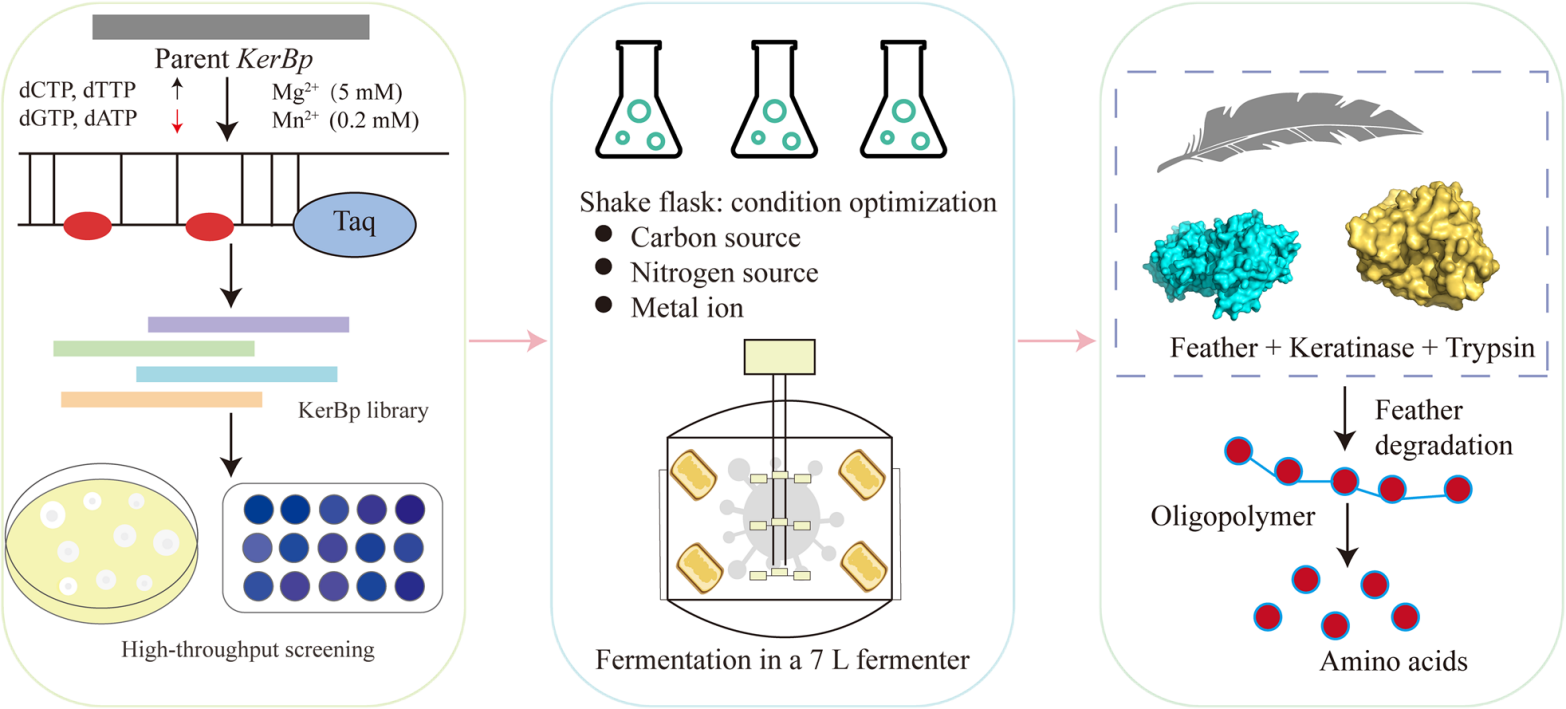

## Introduction

Keratin is a class of insoluble hard protein, widely existing in organism tissues, and composing feathers, hair, wool, nails, horns, hooves and scales (Coulombe and Omary 2002; Wang et al. 2016; Wu et al. 1982). It is reported that nearly 10 million tons of feathers are discarded as by-products every year, and 90% of the discarded feathers are excellent protein resources available for feed industry (Choudhury et al. 2020). Traditional feather degradation methods, such as physical expanding with high temperature and pressure, or chemical dissolution with strong acid and alkali, not only destroy the quality of amino acid and polypeptides, but also consume huge amounts of energy and cause serious environmental pollution. Enzymatic degradation of feather in an environmentally friendly way has a high specificity and efficiency, attracting an increasing attention worldwide (Qiu et al. 2020).

Keratinase is a specific protease produced by microorganisms that exclusively degrades keratin waste, such as wool, feathers, bovine horn and so on. The keratinase-producing microorganisms are mainly bacteria, fungi and actinomycetes, most of which are screened from feather or hair piles (Bokveld et al. 2021; Cao et al. 2021). Among bacteria, the dominant keratinase-producing strain is *Bacillus*, such as *Bacillus licheniformis* PWD-1 that was firstly reported to be able to secrete keratinase to degrade feathers (Lin et al. 1995). In addition, *B. subtilis* (De Paiva et al. 2018), *B. pumilus* (Jagadeesan et al. 2020; Sun et al. 2021) and *B. cereus* (Rajesh et al. 2016) also produce keratinase to degrade keratin waste. Since most of the *Bacillus* are food safety strains, keratinases derived from these strains have the advantages of food safety and convenient for application. It has become a research hotspot in the feed (Liang et al. 2021), leather textile (Zhang et al. 2016), washing and cleaning (Gong et al. 2015), medical (Ye et al. 2020), and cosmetics (Yeo et al. 2018). Meanwhile, studies have shown that the nitrogen source transformed by keratinase degradation of feather waste can be used as fertilizer and soil amendment to promote plant growth (Bhange et al. 2016).

A key step in realizing its industrial applications is to achieve the mass production of keratinase. A variety of expression systems have been used to produce keratinase (Ding et al. 2020; Dong et al. 2017). However, the poor performances of activity and stability still largely limit its commercial interest. Protein engineering methodologies, such as directed evolution and rational design have been widely used for improving the thermostability and specific activity of keratinase. Rational design is based on analyzing the exact structure of available proteins. For example, the activity of keratinase KerBp from *Bacillus pumilus* was increased fivefold by using pro-peptide engineering and saturation site-directed mutation (Su et al. 2017, 2019). Similar results were obtained by Peng et al. (2021). The activity of keratinase KerZ1 from *Bacillus licheniformis* BBE11-1 was improved by 86% using same strategies and the mutant degraded over 90% of the feather into amino acids and oligopolymer. These achievements laid the foundation for the degradation of feathers to make up for the lack of protein resources. Directed evolution, including error-prone PCR, DNA shuffling, annealing oligonucleotide gene rearrangement, cross-extension and non-homologous sequence protein recombination, has been developed and achieved many successes in recent years. Zhao et al. enhanced the half-life of subtilisin E from *B. subtilis* at 65 °C more than 200 times by using directed evolution technique (Zhao and Arnold 1999). Among the types of directed evolution, error-prone PCR has great impact on enzyme catalytic specificity, optimal pH, stability, and substrate specificity and has been widely utilized to screen high-expression strains.

Due to the lack of knowledge about the structure–function relationship of keratinase, error-prone PCR was used to introduce random mutations into keratinase *kerBp* gene in this study. Mutant strains with significantly improved enzyme activity were obtained through high-throughput screening. Then, the yield of keratinase was further increased using high-density fermentation strategy, laying a favorable foundation for the scale preparation and application of keratinase. Finally, the prepared keratinase was employed to degrade feather wastes, and the optimal conditions for enzymatic hydrolysis were explored to provide a theoretical basis for the efficient reuse of feathers and improve the protein utilization in poultry farming.

## Materials and methods

### Strains, plasmids, and media

The original keratinase gene *kerBp* was mined and expressed in our previous studies (Su et al. 2017). *B. subtilis* WB600, *Escherichia.coli* JM109, and the vector pMA5 used in this experiment were all preserved in our laboratory.

The seed medium composition for *E.coli* and *B. subtilis* (g/L): tryptone 10, yeast extract 5, NaCl: 10. The fermentation medium composition for *B. subtilis* (g/L): glycerin 5, yeast extract 24, tryptone 12, K_2_HPO_4_ 12.54, KH_2_PO_4_ 2.31. The initial pH of the medium was natural pH. The optimized medium for *B. subtilis* (g/L): glucose 10, soyabean 18, soybean cake power 60, K_2_HPO_4_ 12.54, KH_2_PO_4_ 2.31.

### Error-prone PCR and expression of keratinase

The gene *kerBp* was amplified with upstream primer (5′-CGG GAT CCA TGT GCG TTA AAA AGA AAA ATG TTA TGA CAA G-3′) and downstream primer (5′-GCA CGC GTT TAA TTT GAT GCT GCT TGC ACA TTA ATC-3′). The plasmid pMA5 was extracted and double-digested with the restriction enzyme *Mlu* I and *Bam *H. I. A randomly mutation library was constructed according to Zhang et al. (Zhang and Zhang 2011) with modifications by error-prone PCR reaction (5 mM MgCl_2_, 0.2 mM MnCl_2_, 0.2 mM dATP, 0.2 mM dGTP, 1 mM dCTP, 1 mM dTTP, 0.05 U/μL polymerase, and 0.4 mM each of the primers). The error-prone PCR was conducted by using the NEB *Taq* DNA polymerase (95 °C denaturation, 3 min; 29 cycles of 95 °C denaturation, 30 s; 57 °C annealing, 30 s; and 72 °C extension, 1.5 min, followed by 72 °C extension for 5 min). The error-prone PCR products were gel-purified and connected to the plasmid pMA5, which were tramsformed into *E. coli* JM109 competent cells. Plasmids obtained were finally expressed in *B. subtilis* WB600 and the strains with large transparent circles were selected directly. The mutant strains were cultured at 37 °C in 250-mL flasks containing 30 mL TB medium (50 µg/mL Kan^r^) for 60 h and the supernatant was collected by centrifuging at 4 °C and 8000*g* for 20 min for keratinase activity measurement.

### Enzymatic properties of mutant keratinase

The optimum temperature of keratinase was determined by measuring enzyme activity at different temperatures (40, 45, 50, 55, 60, 65 and 70 °C). To evaluate thermal stability, the properly diluted keratinase solution was treated at series temperatures for 30 min and cooled on ice. The residual keratinase activity was determined at 50 °C according to the standard enzyme activity method, and the enzyme activity of the untreated enzyme solution was taken as the control. The optimum pH of keratinase was examined with the keratin substrate and enzyme solution appropriately diluted in series pH buffer (pH 6.0, 7.0, 8.0, 9.0, 10.0, 11.0, 12.0).

### Homology modeling of keratinase KerBp

The 3D structure of keratinase KerBp was homologous modeled with the crystal structure of subtilisin NAT (3VYN) from *Bacillus subtilis* (76% sequence similarity), subtilisin BPN (1TO2) from *Bacillus amyloliquefaciens* (76% sequence similarity), and subtilisin DY (1BH6) from *Bacillus licheniformis* (71% sequence similarity) as templates using Discovery Studio. Ramanchandran plot and profile-3D evaluation model were used to verify the rationality of the protein structure.

### Scale-up production of keratinase in 7-L fermenter

Single colony of *Bacillus subtilis* with transparent circle was selected on LB-milk solid plate and inoculated with LB liquid medium for 12 h at 37 °C. 1 mL of the above seed solution was inoculated in 50 mL LB medium and cultured to OD_600_ 0.6–0.8. The secondary seeds were transferred to a 7-L fermenter (Ependorff) with 5% inoculation for high-density fermentation, and kanamycin sulfate was added to 50 μg/mL finally. The speed of fermenter was set as 500 rpm and the temperature was 37 °C. 50% glacial acetic acid or 50% ammonia water was used to adjust the pH value around 7.5. The dissolved oxygen concentration at the initial stage of fermrntation is maintained at 20–30% by adjusting the speed. During the fermentation process, samples were taken periodically to detect bacterial concentration and enzyme activity. When a sudden increase in dissolved oxygen was detected, 50% glucose was added at a flow acceleration rate of 39.53 mL/h.

### Feather degradation

The feather waste used for degradation was collected from a poultry farm (Wuxi, China). The feather degradation experiments were carried out in a 500-mL flask containing 50 mL enzyme solution and 10 g/L chicken feather waste. The prepared keratinase was applied to feather degradation, combining with papain, pepsin, trypsin, bromelain, flavor protease, neutral protease and alkaline protease. The optimal combination of enzymes was selected to degrade feathers and the degradation conditions of compound enzymes were optimized, including proportion of combined enzyme (The enzyme activity radio of trypsin and keratinase were 1:3, 1:2, 1:1, 2:1, 3:1, respectively), enzyme content (800, 1200, 1600, 2000, 2400, 2800 U/mL), temperature (30, 35, 40, 45, 50, 55 °C), pH (7.0, 7.5, 8.0, 8.5, 9.0, 9.5, 10.0), degradation time (8, 16, 24, 32, 40, 48 h) and sulfite content (0.1%, 0.5%, 1%, 1.5%, 2.0%, 2.5%).

### Analytical methods

#### Keratinase assay

Add 100 μL 1% keratin substrate solution to 100 μL appropriately dissoluted keratinase solution, and then incubated at 50 °C for 20 min. Immediately after the reaction, add 200 μL 5%(W/V) TCA to stop the reaction. The control group was added with 200 μL TCA followed by 100 μL keratin substrate solution. Then, the processed samples were centrifuged at 12,000 rpm for 5 min. 200 μL supernatant was mixed with 1 mL 0.4 M Na_2_CO_3_ and 200 μL folinol solution, and placed in a 40 °C water bath for 20 min. The absorbance value was detected at 660 nm.

#### Feather degradation rate

The degradation products were filtered to leave the undegraded feathers, which then were washed with deionized water three times to completely remove the soluble substances and thallus, and dried at 65 °C for 24 h to constant weight. Degradation rate of feathers is defined as the change in dry weight before and after degradation.

#### Reducing power analysis of keratinase

Two different methods were used to evaluate the reducibility of keratinase. Reducing power was evaluated as the ability to reduce Fe^3+^ to Fe^2+^, using the potassium ferricyanide reduction method (Clerici et al. 2021). The diluted keratinase (300 μL) was mixed with 0.2 M PBS (300 μL) buffer solution (pH 6.6) and 300 μL 1% (W/V) potassium ferricyanate. After incubation at 50 °C for 20 min, 300 μL 10% (w/v) TCA was added to stop the reaction, and centrifuged at 4000 rpm for 10 min. Then 200 μL supernatant was mixed 800 μL 0.01% (W/V) ferric chloride solution and incubated at 30 °C for 10 min. Finally, the absorbance of the reaction system was detected at 700 nm. The value of the absorbance represents the level of reducing power.

2,2′-Azo-bis-(3-ethylbenzothiazoline)-6-sulfonic acid (ABTS) radical scavenging experiment is mainly based on Re et al. (1999). Add 10 μL of culture supernatant to 1 mL of ABTS radical working solution and messure the absorbance at 734 nm after 6 min. In the control group, 10 μL of distilled water was added to replace the culture supernatant. The ABTS radical scavenging is calculated as follows:

$${\text{Scavenging}}\,(\% ) = \left[ {{{\left( {{\text{Abs}}_{{{\text{control}}}} - {\text{Abs}}_{{{\text{sample}}}} } \right)} \mathord{\left/ {\vphantom {{\left( {{\text{Abs}}_{{{\text{control}}}} - {\text{Abs}}_{{{\text{sample}}}} } \right)} {{\text{Abs}}_{{{\text{control}}}} }}} \right. \kern-\nulldelimiterspace} {{\text{Abs}}_{{{\text{control}}}} }}} \right] \times 100.$$

#### Analysis of amino acids

The supernatant of degradation products was added with the same volume of TCA. After centrifugation for 30 min, the supernatant passed through a 0.2-μm membrane filter. The free amino acid composition was determined by high performance liquid chromatography (HPLC, Agilent 1260, Santa Clara, CA, USA) with *o*-phthalaldehyde-9-fluorovinyl methyl chloroformate (OPA-FMOC) pre-column derivatization. An Agilent spectroscopy system was used to calculate the concentration from the peak area obtained. The mobile phase is acetonitrile–methanol. The detector is VWD, the wavelength is 338 nm, and the flow rate is 1 mL/min. The chromatographic column is Hypersil ODS-2 (250 × 4.6 mm, 5 μm), the temperature is 40 °C, and the injection volume is 10 μL.

#### Analysis of soluble peptides

Based on the comparison of peak time and peak area, the molecular weight distribution of soluble peptides was determined by high performance liquid chromatography (Agilent 1260, USA). The samples were centrifuged at 8000 rpm for 5 min, and then the supernatant was collected and passed through a 0.2-μm membrane filter. The molecular weight distribution of soluble peptides was obtained by gradient elution on TSK gel G2000SWXL (7.8 × 300 mm) using phosphate buffer as mobile phase. The detector, wavelength and flow rate were VWD, 214 ηm and 0.8 mL/min, respectively (Peng et al. 2019).

### Statistical analysis

All assays in this study were performed in triplicate. Data processing in this study was performed by using the mean standard deviation (± SD) and analyzed via GraphPad Prism 7 (San Diego, CA, USA).

## Results and discussion

### Directed evolution of keratinase by error-prone PCR

**Fig. 1:**
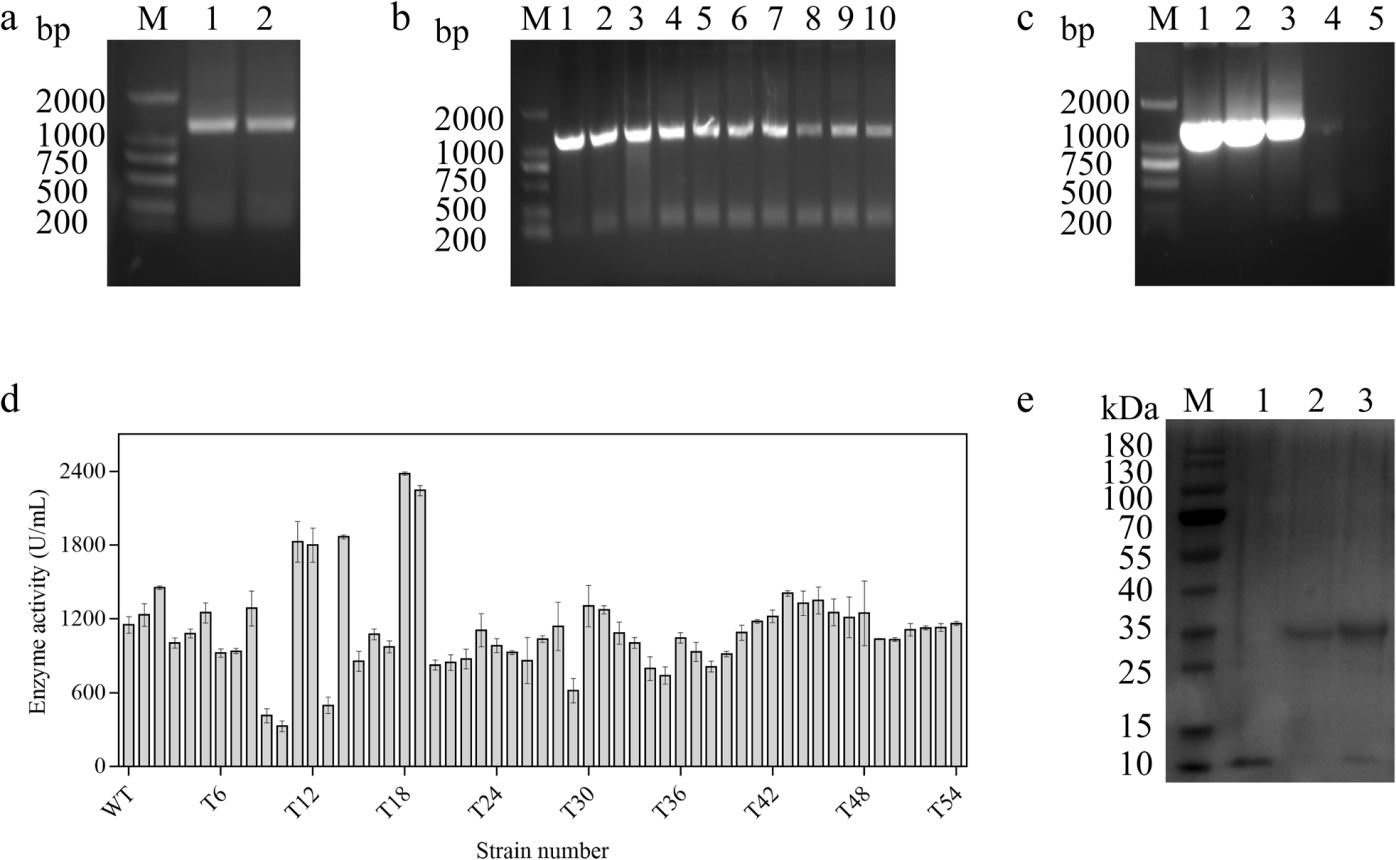
Screening of recombinant strains. **a** Verification of gene *kerBp*. Lane M, DNA marker; Lane 1–2, PCR amplification of *kerBp*. **b** Mg^2+^ concentration gradient. Lane M, DNA marker; lane 1–10, Mg^2+^: 1 mM, 2 Mm, 3 mM, 4 mM, 5 mM, 6 mM, 7 mM, 8 mM, 9 mM, 10 mM. **c** Mn^2+^ concentration gradient. Lane M, DNA marker; lane 1–5, Mn^2+^: 0.1 mM, 0.2 Mm, 0.3 mM, 0.4 mM, 0.5 mM. **d** Rescreening results of shake flasks. **e** SDS-PAGE analysis of WT and variant T18. Lane M, protein MW markers; lane 1, supernatant of fermentation broth from *B. subtilis* WB600 harboring pMA5 as the control; lane 2, WT; lane 3, variant T18

Directed evolution is an efficient tool to generate variants with fresh or enhanced properties. Error-prone PCR was adopted in this study to construct mutant libraries and provide superior keratinases for better degradation performance. Figure 1a shows a 1200-bp band of error-prone PCR products, consistent with the target gene *kerBp*. Moreover, it was found that the efficiency of error-prone PCR was affected by changing the concentration of Mg^2+^ (Fig. 1b) and Mn^2+^ (Fig. 1c). With the increase of metal ions concentration, the efficiency was decreased gradually and less product was obtained; while a low concentration of metal ions leading to a low base mutation rate. According to the experimental results, 5 mM Mg^2+^ and 0.2 mM Mn^2+^ were selected as the experimental condition. As a result, a library with more than 8000 mutants was constructed after two rounds of error-prone PCR and high-throughput screened by fluorescence chromogenic enzyme activity determination. As expectation, nine strains with increased enzyme activity were selected (Table 1), among which, the highest enzyme activity of the mutant T18 (R72S/F107Y/N291S/N295D) was 2382 U/mL, that was 2.1 times of the original keratinase activity (Fig. 1d). The SDS-PAGE results (Fig. 1e) indicated an expected keratinase protein band.

### Enzymatic properties of mutant keratinase

**Fig. 2:**
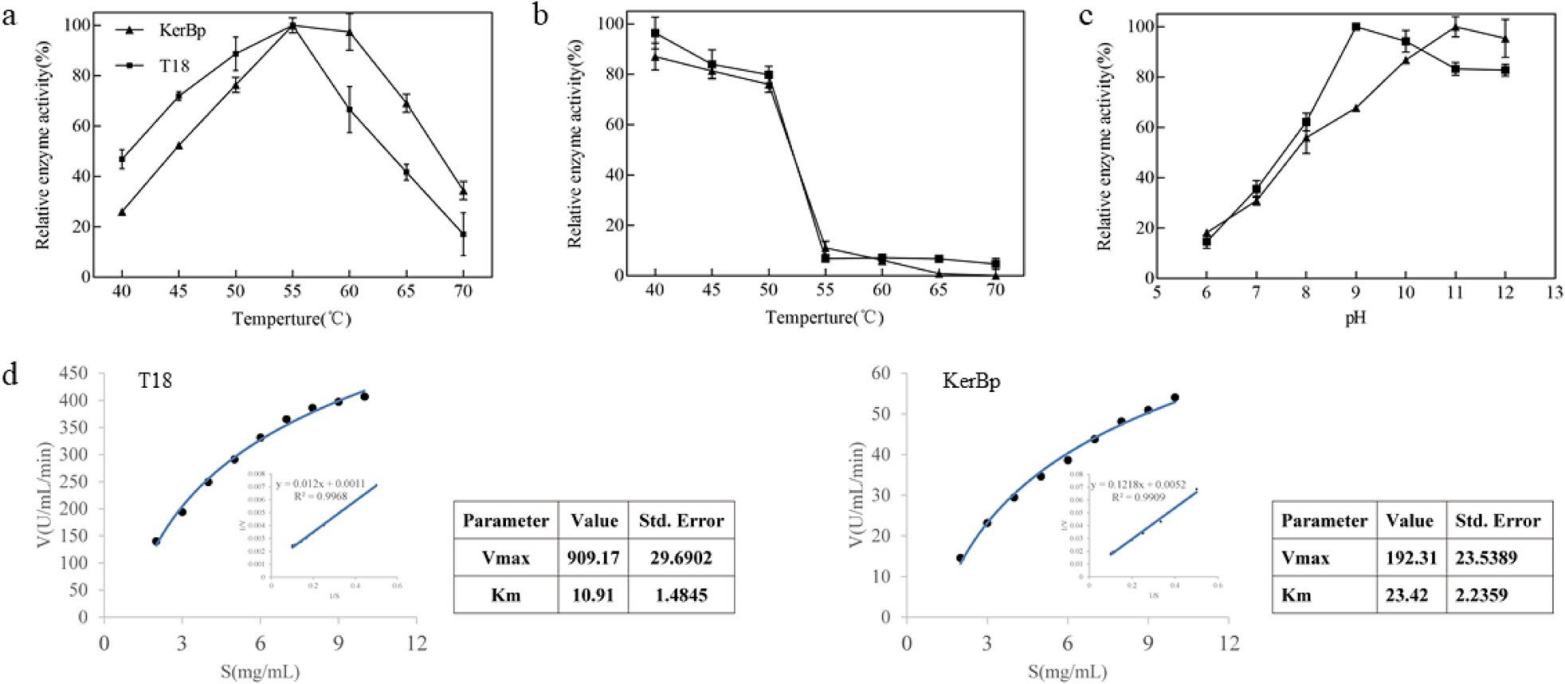
Enzymatic properties of variant T18. **a** Optimum temperature; **b** thermal stability; **c** optimum pH; **d** steady-state kinetic analysis

Due to the uncertainty of error-prone PCR, not only the enzymatic activity of mutant is altered, but other properties such as thermostability and optimum pH may be also affected. Thus, the effects on catalytic properties were determined. The highest specific activity is defined as 100%. As shown in Fig. 2a, the specific activity of keratinase was the highest at 55 °C before and after the mutation, indicating that the optimal temperature of keratinase was not affected by mutation. But the mutant keratinase activity remained over 70% in the range of 45–55 °C, while the original keratinase activity decreased to 52%. In terms of thermal stability (Fig. 2b), the remaining enzyme activity of the mutant keratinase is higher than the original enzyme activity in the range of 40–50 °C. As shown in Fig. 2c, the specific activity of the mutant enzyme was the highest at pH 9, while the optimal pH of the original keratinase was 11, indicating that the optimal pH of the keratinase was changed after mutation. The decrease of the optimum pH provides a suitable pH environment for the complexation of keratinase and other proteases in the later stage.

In the past 20 years, the directed evolution of enzymes has gradually become a research hotspot in the field of bio-catalysis. Compared with natural enzymes, engineered enzymes obtained through directed evolution show enhanced properties such as higher activity, higher thermostability, better stereoselectivity and better alkaline or acid stability. Li et al. (2021) obtained a variant (G95P) with ninefold enhancement in specific activity by error-prone PCR. In addition, the thermostability and alkaline stability of the alkaline protease were also enhanced. In this study, directed evolution technology was used to modify keratinase, which not only increased the activity of keratinase, but also improved the thermostability of keratinase. The improved enzyme activity, thermal stability and the decrease of optimal pH make the mutant keratinase more suitable for industrial applications, especially for degradation of feathers by combining keratinase with other proteases, which would improve the hydrolysis efficiency of feathers.

### Homologous modeling analysis

**Fig. 3:**
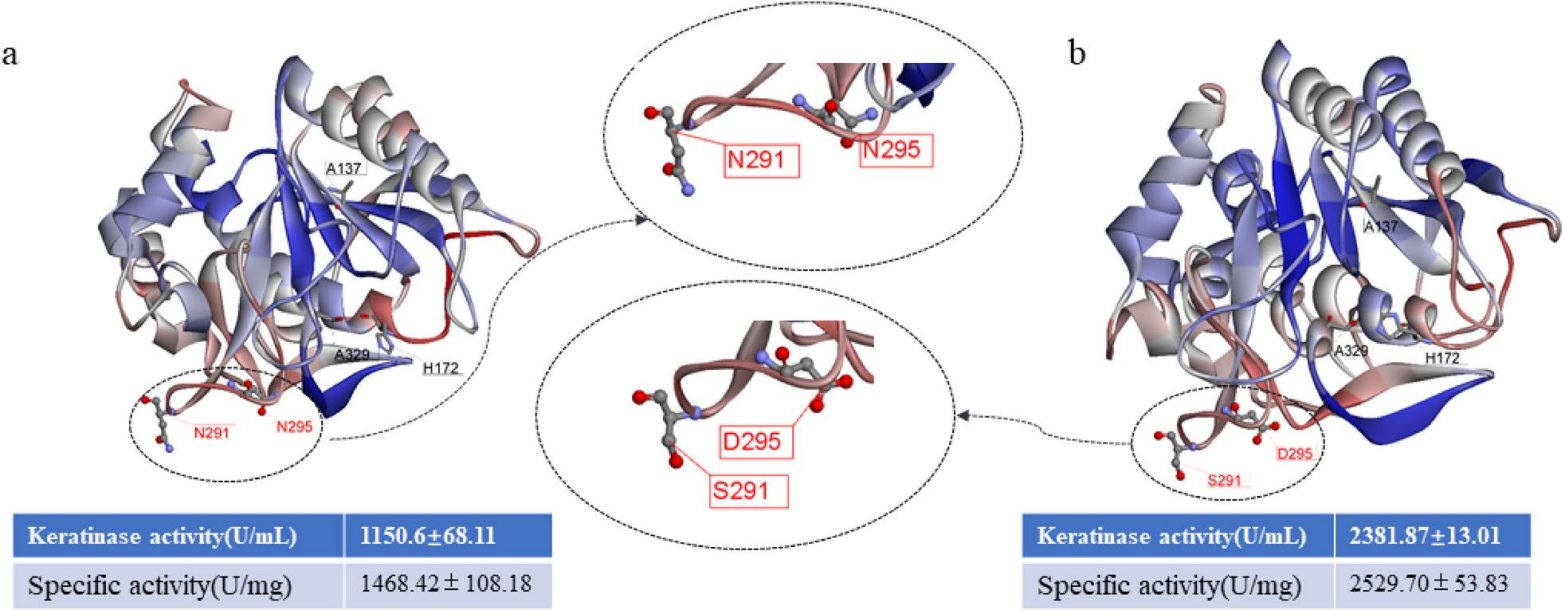
Three-dimensional structural model by homology modeling. **a** KerBp. **b** Variant T18

The structure modeling of keratinase KerBp was predicted. The framework was consisted with ten *β*-folds and eight *α*-helixes, as shown in Fig. 3. Two mutation sites of R72S/F107Y are located in the non-conserved region of pro-peptide region and the change in amino acid hydrophobicity affects spatial configuration of the pro-peptide, leading to folding and degradation of the mature peptide. Studies have shown that mutations at appropriate site of pro-peptide can change the folding speed of the protein and increase the activity and production of extracellular enzyme, so the changes on pro-peptide promoted the increase of keratinase activity (Grimsby et al. 2010). In our previous study, Su et al. (2019) carried out saturation mutations at six potential sites in the pro-peptide region of keratinase, and the activity of keratinase increased from 179 to 1114 U/mL. The other two mutation points of N291S/N295D are located on the mature peptide, and N291/N295 is close to S1 pocket region. The structure of the S1 and S4 substrate pockets has an important influence on the substrate specificity of protease (Fang et al. 2015). The change of amino acids may affect S1 pocket structure, thus enhancing the substrate specificity of keratinase. The application of directed evolution for molecular modification in enzyme catalysis has achieved favorable results (Buller et al. 2018). Directed evolution can modify the catalytic pocket of the enzyme (Laksmi et al. 2020) and improve its substrate specificity (Dai et al. 2021; Zhou et al. 2021).

### High-density fermentation

**Fig. 4:**
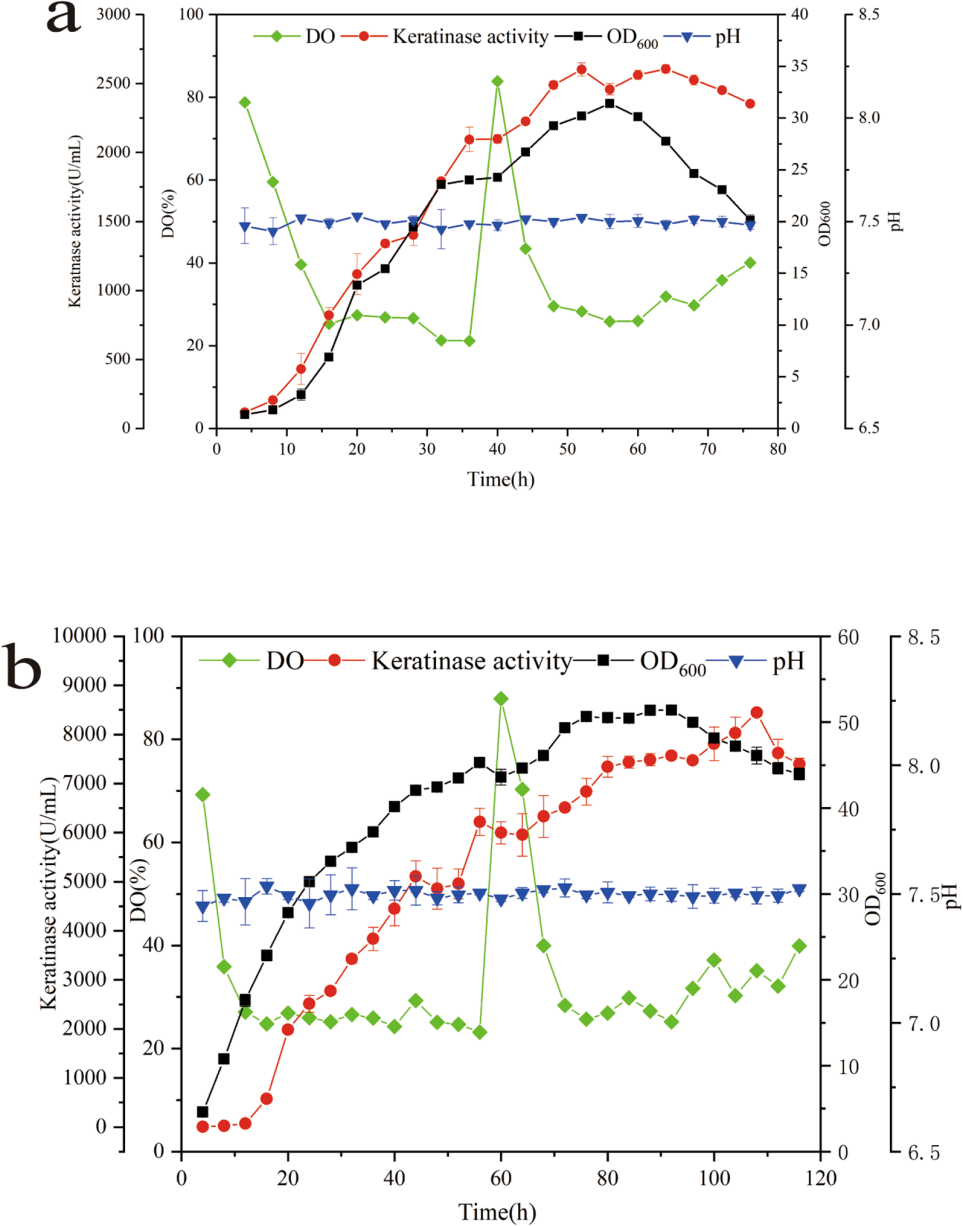
High-density fermentation of the variant T18 and the original strain in a 7-L fermenter. **a** WT; **b** variant T18

To measure the growth and production of mutant keratinase and explore its industrial potential, the fermentation was carried out in a 7-L fermenter. According to the regular detection of cell density and enzyme activity as shown in Fig. 4, the mutant strain grew logarithmically within 0–16 h, while the enzyme activity gradually increased. During 16–60 h, the cells continued to grow and tended to be stable. At this stage, keratinase was continuously produced and accumulated, and the enzyme expression level continued to increase. Subsequently, the cells continued to grow through streaming medium and reached the maximum concentration at 92 h with OD_600_ value of 51.41. The enzyme activity continued to accumulate and reached the maximum with 8448 U/mL at 108 h.

There are several strategies to improve the activity and yield of recombinant keratinase, such as promoter engineering (Gong et al. 2020), signal peptide engineering (Tian et al. 2019), heterologous expression (Jin et al. 2019; Yong et al. 2020), site-directed mutagenesis (Jaouadi et al. 2014) and pro-peptide engineering (Su et al. 2019). Studies have shown that fermentation optimization is one of the most important strategies to improve keratinase activity and yield. The fermentation conditions of the neutral protease derived from *B. subtilis* were optimized, and the enzyme activity was increased by nearly 2.8 times (He et al. 2021). Using waste feathers as carbon source, the production of keratinase was increased by five times using deep liquid fermentation optimization method (Jana et al. 2020).

**Table 1:** Comparison of the performance indexes of mutant strains and WT

Enzyme	Keratinase activity (U/mL)	Specific activity (U/mg)	Degradation rate (%)
T1	1231.07 ± 91.97	1189.79 ± 77.24	36.84 ± 0.88
T3	1453.13 ± 13.95	1577.74 ± 76.78	24.52 ± 1.71
T5	1248.92 ± 82.58	1215.74 ± 158.08	34.48 ± 1.77
T8	1285.2 ± 142.63	1050.93 ± 101.98	25.78 ± 1.80
T11	1827.93 ± 165.46	1723.44 ± 200.56	40.42 ± 1.60
T12	1800 ± 137.61	1746.34 ± 92.22	42.04 ± 1.34
T14	1866.33 ± 16.58	1515.63 ± 15.90	37.68 ± 0.96
T18	2381.87 ± 13.01	2529.70 ± 53.83	51.42 ± 0.62
T19	2243.87 ± 41.80	1968.43 ± 122.99	40.2 ± 0.38
WT	1150.6 ± 68.11	1468.42 ± 108.18	33.57 ± 2.13

### Feather degradation

**Fig. 5:**
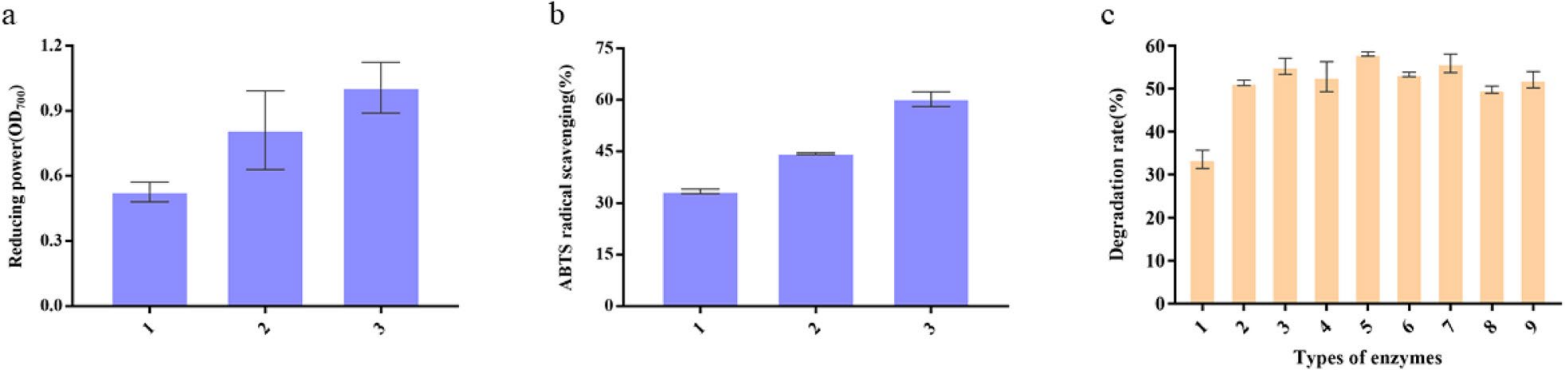
Comparison of reducing power and degradation of feathers. **a** Reducing power (OD_700_). 1, pMA5; 2, WT; 3, variant T18. **b** ATBS radical scavenging. 1, pMA5; 2, WT; 3, variant T18. **c** Degradation of feathers by combined enzymes. 1, WT; 2, variant T18; 3, papain and variant T18; 4, pepsin and variant T18; 5, trypsin and variant T18; 6, bromelain and variant T18; 7, flavor protease and variant T18; 8, neutral protease and variant T18; 9, alkaline protease and variant T18

Although the degradation mechanism of keratinase is still not fully understood, it is widely recognized that reducing power plays an important role in the degradation process (Lange et al. 2016; Ramnani et al. 2005). Herein, the reducing ability of keratinase was assured by reduction of Fe^3+^ (Fig. 5a) and ABTS radicals scavenging test (Fig. 5b). Reducing power measurement is based on Prussian blue production as an indicator. The samples reduce potassium ferricyanide, and then use ferrous ions to generate Prussian blue. The value of the absorbance at 700 nm represents the level of reducing power with a positive relationship between them. It was found that the keratinase of the mutant strain had a stronger reducing power than the control. This unique property indicates that the keratinase of the mutant strain has high reducibility and could be used in feather degradation reaction. Compared with the control pMA5 and the original keratinase, the mutant keratinase scavenged more than 60% of ABTS radicals within 6 min, showing stronger antioxidant activity and better degradation performance.

**Fig. 6:**
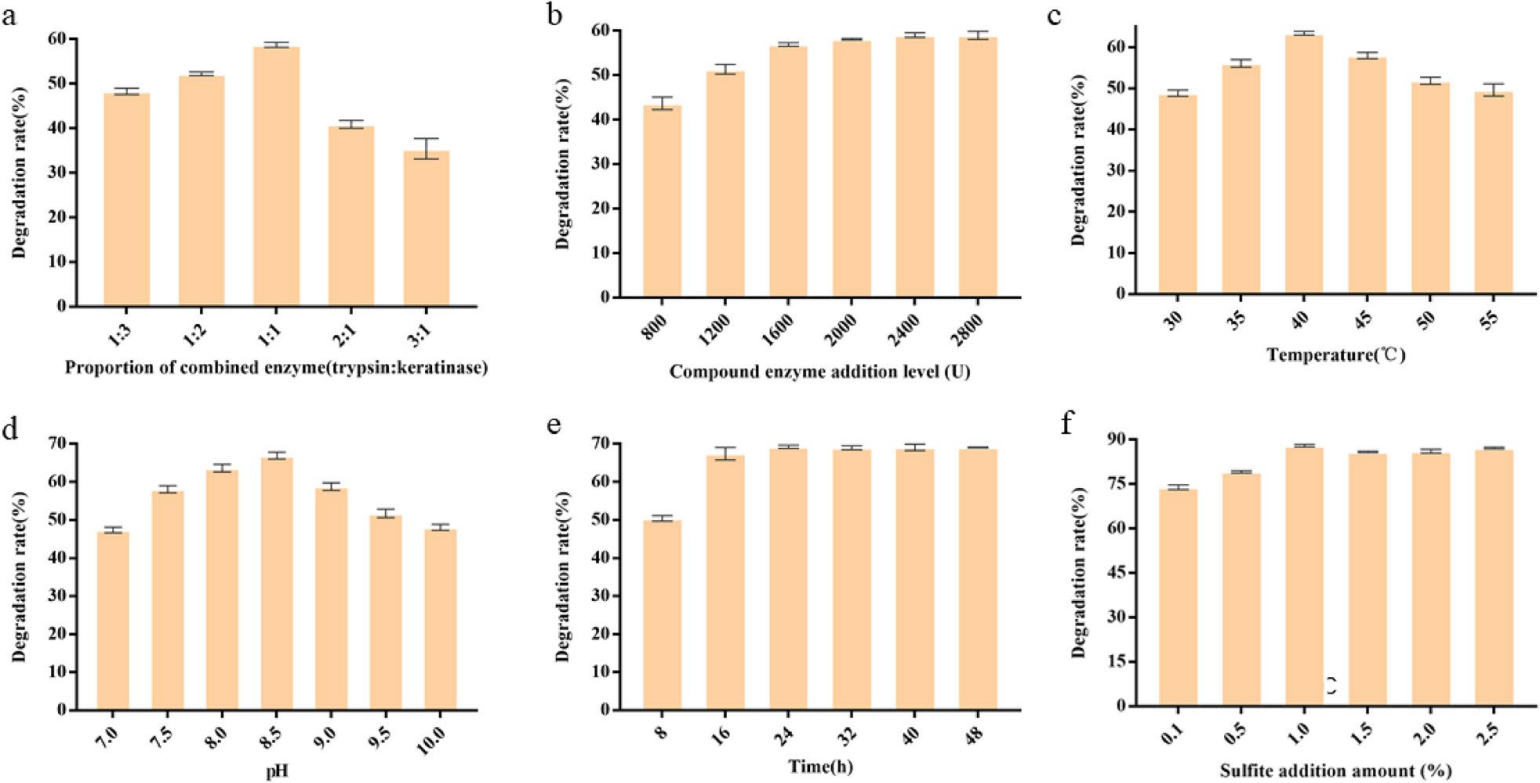
Optimization of compound enzyme conditions. **a** Proportion of compound enzyme (trypsin: keratinase); **b** compound enzyme addition level; **c** temperature; **d** pH; **e** time; **f** sulfite addition amount

**Fig. 7:**
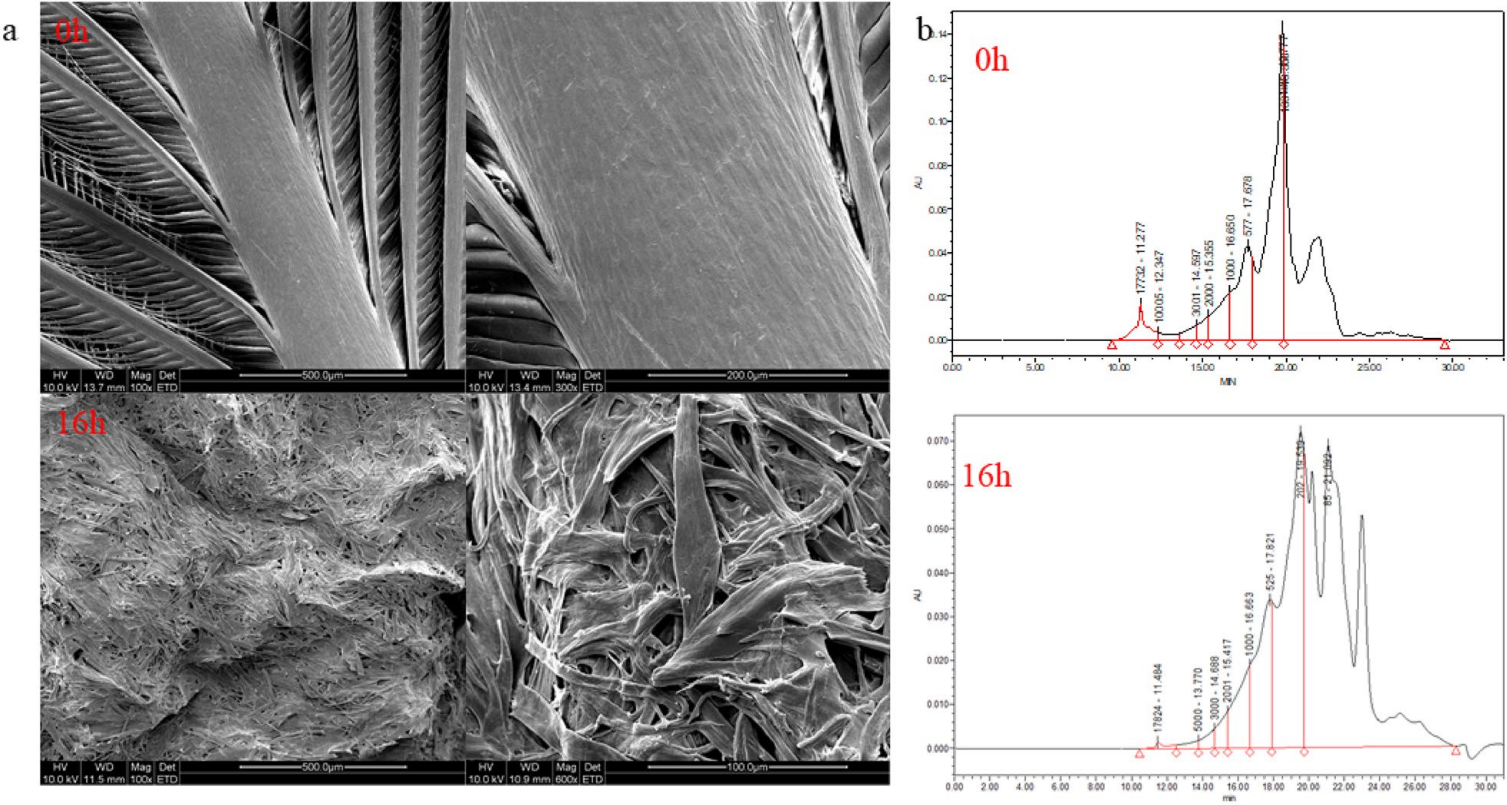
Scanning electron microscopy and molecular weight distribution of the degradation products. **a** Scanning electron microscopy; **b** molecular weight distribution of polypeptide

Since the mutant keratinase had both reducing and hydrolyzing properties, when the keratinase was used alone to degrade feathers, the degradation rate reached 49% (Fig. 5c). In order to further improve the degradation rate of feathers, the keratinase was compounded with trypsin and the degradation conditions were optimized, by which degradation rate increased from 49 to 89% (Fig. 6). The SEM on the structure of degraded feathers (Fig. 7a) showed that, compared with the blank control group, after 16 h of hydrolysis, barbs, barbules and accessory pinna were completely separated, and the scapus broke irregularly with damage appeared on the surface. The ability of keratinase to degrade feathers significantly in a short period provides the basis for its potential application in bioconversion of keratin wastes into valuable protein resources. Table 2 indicates the change of amino acid concentration in feather degradation products during feather degradation. After hydrolysis for 12 h, the total content of amino acids in the degradation solution reached up to 4972.34 mg/mL, which was higher than those reported previously (Table 3). The concentrations of histidine (His), glycine (Gly), threonine (Thr), tyrosine (Tyr) and lysine (Lys) increased by 248.7 mg/L, 197.38 mg/L, 911.82 mg/L, 241.41 mg/L and 783.42 mg/L, respectively. Threonine increased by 174 times from the initial value at 12 h of degradation. Threonine is the second and third limiting amino acid in pig and poultry feed, respectively. It helps to adjust the amino acid balance, promotes the growth of livestock, improves the nutritional value and amino acid digestibility, and reduces the cost of feed materials. Therefore, hydrolyzed feathers have great potential in the production of feed additives and amino acids. Moreover, the soluble peptides in the degradation solution were analyzed. As shown in Fig. 7b, the molecular weight of peptides in the hydrolysate gradually decreased, and the molecular weight of peptides in the hydrolysate was concentrated below 1 kDa, indicating that the degradation products were mainly composed of oligopolymer. These oligopolymer are easily utilized by animals and have application signification in feed industry (Table 3).

**Table 2:** The changes of amino acid concentration during feather degradation process by the variant T18

Amino acid	Concentration (mg/L)			
	0 h	12 h	24 h	36 h
asp	25.09 ± 0.31	44.05 ± 1.47	28.99 ± 1.80	23.71 ± 4.58
glu	41.12 ± 0.16	122.94 ± 3.16	11.85 ± 2.63	17.32 ± 4.12
ser	0.88 ± 0.80	12.99 ± 0.66	1.92 ± 1.32	0.80 ± 0.59
his	21.03 ± 1.82	269.73 ± 0.45	23.01 ± 1.70	17.31 ± 4.78
gly	22.16 ± 0.13	219.54 ± 6.42	56.85 ± 7.11	3.18 ± 0.27
thr	5.27 ± 0.21	917.09 ± 24.37	272.72 ± 5.64	537.47 ± 12.29
arg	11.31 ± 0.56	25.09 ± 0.43	0.58 ± 0.042	1.06 ± 0.51
ala	32.5 ± 0.62	23.80 ± 0.14	2.61 ± 0.70	3.64 ± 0.42
tyr	146.01 ± 2.09	387.42 ± 5.94	121.41 ± 4.24	114.32 ± 7.87
cys	6.38 ± 0.69	14.56 ± 1.47	4.84 ± 0.67	4.26 ± 0.50
val	165.18 ± 0.83	389.84 ± 22.32	22.86 ± 12.06	22.66 ± 2.60
met	116.92 ± 2.81	370.51 ± 15.36	72.61 ± 6.84	44.6 ± 6.52
phe	199.02 ± 0.95	688.07 ± 14.47	152.44 ± 14.56	154.41 ± 1.88
ile	63.84 ± 0.88	55.39 ± 1.64	1.80 ± 0.30	1.23 ± 0.24
leu	180.32 ± 1.63	349.90 ± 8.14	7.16 ± 1.28	6.57 ± 0.93
lys	282.93 ± 5.09	1066.35 ± 13.87	187.07 ± 6.01	149.06 ± 13.82
pro	231.74 ± 12.78	14.43 ± 1.70	113.69 ± 16.87	111.81 ± 12.49

**Table 3:** Comparison of amino acids in feather degradation products

Amino acid	Concentration (mg/L)			
	WT (12 h)	Variant T18 (12 h)	KerSMD and KerSMF (48 h) (Peng et al. 2019)	*Bacillus pumilus* AR57 keratinase (Jagadeesan et al. 2020)
asp	64.66 ± 1.84	44.05 ± 1.47	15.55 ± 1.09	0.612
glu	245.91 ± 6.95	122.94 ± 3.16	20.19 ± 0.13	1.242
ser	10.00 ± 1.08	12.99 ± 0.66	14.61 ± 0.18	9.805
his	44.05 ± 0.39	269.73 ± 0.45	ND	4.794
gly	136.19 ± 7.33	219.54 ± 6.42	14.34 ± 0.24	1.421
thr	464.77 ± 56.77	917.09 ± 24.37	15.74 ± 0.22	3.514
arg	8.8 ± 0.61	25.09 ± 0.43	22.13 ± 0.49	14.110
ala	504.30 ± 5.88	23.80 ± 0.14	10.05 ± 0.28	0.517
tyr	195.87 ± 0.89	387.42 ± 5.94	171.53 ± 0.42	18.789
cys	10.82 ± 1.49	14.56 ± 1.47	ND	ND
val	148.99 ± 8.97	389.84 ± 22.32	207.51 ± 0.29	8.716
met	187.58 ± 11.44	370.51 ± 15.36	0 ± 0.41	7.684
phe	348.35 ± 1.36	688.07 ± 14.47	183.73 ± 0.21	33.153
ile	18.99 ± 0.94	55.39 ± 1.64	72.96 ± 0.49	4.749
leu	91.94 ± 1.88	349.90 ± 8.14	126.61 ± 0.73	7.083
lys	510.48 ± 5.08	1066.35 ± 13.87	20.94 ± 0.73	3.772
pro	28.14 ± 2.86	14.43 ± 1.70	ND	ND

## Conclusions

In this study, a keratinase mutant with improved activity was obtained via directed evolution technique employing error-prone PCR and high-throughput screening. Through high-density fermentation, the enzyme activity increased from 1150 to 8448 U/mL. Moreover, in order to understand the catalytic function of the existing keratinase in depth, the homologous structure of the three-dimensional structure was modeled, and the keratinase was characterized. The keratinase after mutation had better temperature stability and optimum pH suitable for compounding. In addition, the combination of keratinase and trypsin degrades feathers, and the product contains a large number of amino acids, short peptides and oligopeptides, which provides protein resources for poultry and has potential applications in the feed industry.

## Data Availability

All data generated or analyzed during this study are included in the main manuscript file.
